# Genome-wide translational response of *Candida albicans* to fluconazole treatment

**DOI:** 10.1128/spectrum.02572-23

**Published:** 2023-08-23

**Authors:** Saket Choudhary, Vasanthakrishna Mundodi, Andrew D. Smith, David Kadosh

**Affiliations:** 1 Quantitative and Computational Biology, University of Southern California, Los Angeles, California, USA; 2 Department of Microbiology, Immunology, and Molecular Genetics, University of Texas Health Science Center at San Antonio, San Antonio, Texas, USA; The University of Iowa, Iowa City, Iowa, USA

**Keywords:** fluconazole, ribosome profiling, translational control, transcriptional profiling, *Candida albicans*

## Abstract

Azoles are commonly used for the treatment of fungal infections, and the ability of human fungal pathogens to rapidly respond to azole treatment is critical for the development of antifungal resistance. While the roles of genetic mutations, chromosomal rearrangements, and transcriptional mechanisms in azole resistance have been well-characterized, very little is known about post-transcriptional and translational mechanisms that drive this process. In addition, most previous genome-wide studies have focused on transcriptional responses to azole treatment and likely serve as inaccurate proxies for changes in protein expression due to extensive post-transcriptional and translational regulation. In this study, we use ribosome profiling to provide the first picture of the global translational response of a major human fungal pathogen, *Candida albicans*, to treatment with fluconazole (Flu), one of the most widely used azole drugs. We identify sets of genes showing significantly altered translational efficiency, including genes associated with a variety of biological processes such as the cell cycle, DNA repair, cell wall/cell membrane biosynthesis, transport, signaling, DNA- and RNA-binding activities, and protein synthesis. We observe both similarities and differences among the most highly represented gene categories (as defined by gene ontology) that are regulated by fluconazole at the translational vs transcriptional levels. Importantly, however, very few genes that are translationally regulated by fluconazole are also controlled transcriptionally under this condition. Our findings suggest that *C. albicans* possesses distinct translational mechanisms that are important for the response to antifungal treatment, which could eventually be targeted by novel antifungal therapies.

**IMPORTANCE:**

Azoles are one of the most commonly used drug classes to treat human fungal pathogens. While point mutations, chromosomal rearrangements, and transcriptional mechanisms that drive azole resistance have been well-characterized, we know very little about the role of translational mechanisms. In this study, we determined the global translational profile of genes that are expressed in the major human fungal pathogen *Candida albicans* in response to fluconazole, one of the most widely used azole drugs. We find both similarities and differences among the most highly represented categories of genes regulated by fluconazole at the transcriptional and translational levels. Interestingly, however, many of the specific genes that are regulated by fluconazole at the translational level do not appear to be controlled by transcriptional mechanisms under this condition. Our results suggest that distinct *C. albicans* translational mechanisms control the response to antifungals and could eventually be targeted in the development of new therapies.

## INTRODUCTION

*Candida albicans* is the fourth leading cause of hospital-acquired bloodstream infections in the United States (1 – 3) and a major human fungal pathogen that is responsible for a diverse array of both systemic and mucosal infections (4 – 8). Cancer patients undergoing chemotherapy, recipients of artificial joints/prosthetic devices, AIDS patients, organ transplant recipients, and additional immunocompromised individuals are susceptible to acquiring a wide variety of both systemic and mucosal infections (4 – 8). The cost of treating patients with hospital-acquired *Candida* infections is approximately $1 billion per year (9, 10).

Currently, only three major classes of antifungals are available to treat patients with candidiasis: azoles, echinocandins, and polyenes (11 – 14). Azoles, the most commonly used drug class, specifically target the ergosterol biosynthesis pathway, which is important for maintaining fungal cell membrane integrity (15). Repeated treatment of recurring *Candida* infections with antifungals, in addition to increased use of long-term antibiotic prophylaxis, has led to a significant increase in the frequency of drug-resistant clinical isolates (11 – 14). Antifungal resistance, and resistance to azoles in particular, is a clinically significant problem and has been classified as a serious health threat by both the Centers for Disease Control and World Health Organization (http://apps.who.int/iris/bitstream/10665/112642/1/9789241564748_eng.pdf, http://www.cdc.gov/drugresistance/threat-report-2013/pdf/ar-threats-2013-508.pdf#page=63).

Many previous studies have characterized genetic point mutations, transcriptional mechanisms, and chromosomal rearrangements that confer azole resistance in *C. albicans* and other human fungal pathogens. For example, drug-resistant isolates have shown increased transcription of *ERG11*, encoding the azole target enzyme lanosterol 14-α-demethylase, or point mutations that reduce the affinity of Erg11 for azoles (15 – 18). While considerably less is known about translational mechanisms that control antifungal resistance, several lines of evidence suggest that they play important roles. In the related human fungal pathogen *Candida glabrata*, Erg11 protein, but not transcript, levels were shown to be up-regulated in azole-resistant vs. susceptible isolates (19). Also in *C. glabrata*, *SSD1*, which encodes a RNA-binding protein and translational inhibitor, has recently been shown to regulate echinocandin resistance as well as expression of the Fks1 and Fks2 echinocandin target proteins (20). In addition, a previous study in *C. albicans* has shown that adaptive mistranslation of the CUG codon to leucine, rather than serine, can specifically promote the acquisition of fluconazole resistance (21).

Eukaryotic 5′ untranslated regions (UTRs) are known to direct translational control via upstream open reading frames as well as by formation of secondary structures, which can associate with RNA-binding proteins to inhibit ribosome accessibility and/or promote stalling; zip code sequences in 5′ UTRs can also direct transcripts to translationally inactive cellular compartments (22 – 24). In *C. albicans*, 5′ UTR-mediated translational efficiency mechanisms are known to control white-opaque switching, mating, and morphology (25 – 27). Several transcriptional regulators of azole resistance, including Upc2 and Fcr1, possess long (>500 bp) 5′ UTR regions and could be under translational control as well (28). Finally, a comparative study has demonstrated that only about one-third of *C. albicans* proteins identified as showing differential protein expression (as determined by proteomics) in fluconazole-resistant vs susceptible isolates also showed altered transcript levels (29), suggesting that there is significant regulation at the post-transcriptional, and possibly translational, level.

Whole-genome transcriptional profiling experiments have reported significant alterations in *C. albicans* transcript levels that occur in response to treatment with azoles (30 – 33). Up-regulated genes included those involved in plasma membrane/cell wall synthesis/maintenance, stress responses, metabolism (including carbohydrate and lipid/fatty acid metabolism), ergosterol biosynthesis, and drug efflux. Genes down-regulated in response to fluconazole were involved in protein synthesis and DNA synthesis/repair. However, whole-genome transcriptional profiling can often serve as an inaccurate proxy for gene expression due to extensive changes at the post-transcriptional and translational levels.

In order to gain a better understanding of global translational changes in *C. albicans* gene expression, we have been using a powerful new technique, ribosome profiling (34). Through genome-wide identification of all sequences bound to ribosomes, ribosome profiling provides a comprehensive picture of the translational activity of every gene and, importantly, also serves to identify certain key translational parameters, including translational efficiency. We have recently used this approach to define the global translational profile of the *C. albicans* morphological transition, a key virulence trait (35). Many transporters and permeases previously demonstrated to be transcriptionally regulated in response to antifungal treatment also showed increased translational efficiency during filamentation, as well as the *YCF1* ABC transporter, likely involved in multidrug resistance, and *ERG251*, involved in ergosterol biosynthesis (30, 35, 36). Interestingly, several genes involved in filamentation and pathogenesis known to be strongly induced at the transcriptional level showed reduced translational efficiency, suggesting that a translational fine-tuning mechanism is in place to tightly control their expression under filament-inducing conditions (35). These findings indicated that *C. albicans* translational expression patterns do not simply parallel transcriptional expression and highlight the importance of ribosome profiling for the identification of novel translational mechanisms. In the current study, we use ribosome profiling to examine the *C. albicans* genome-wide translational profile upon fluconazole treatment. We observe both similarities and differences among the most highly represented categories of genes controlled at the translational vs transcriptional levels. However, very few genes that are regulated at the translational level by fluconazole are also regulated by transcription under this condition. In addition to providing the first report of the global translational profile of a human fungal pathogen in response to antifungal treatment, this study is likely to lead to the identification of novel translational mechanisms that drive drug resistance and can be exploited for the development of new and more effective antifungal therapies.

## RESULTS

### Ribosome profiling of *C. albicans* cells grown in the presence and absence of fluconazole

A wild-type *C. albicans* strain was grown in the presence of 1 µg/mL fluconazole or DMSO (a vehicle-only, no drug control) for 6 h. This concentration and exposure time were selected for our analysis because we were able to consistently observe 50% growth inhibition (IC_50_) specifically in response to fluconazole compared to the vehicle-only control. At the 6-h time point, separate aliquots of cells were harvested for RNA-seq and Ribo-seq analysis. Ribosome profiling was performed using a protocol optimized for *C. albicans* that we have described previously (35).

A read length distribution analysis showed that the large majority of Ribo-seq reads were in the 28–31 nt range, peaking at 30 nt (Fig. S1A), which is characteristic of ribosome-protected fragments (RPFs) in *C. albicans* and other organisms (35, 37). As expected, and as previously observed for our genome-wide translational analysis of *C. albicans* morphogenesis (35), Ribo-seq and RNA-seq read counts showed a strong correlation at the genome-wide level (Fig. S1B). Metagene analysis also showed a clear 3-nucleotide periodicity in the region of the start codon specifically for RPF, but not total RNA, reads (Fig. S1C). A larger peak at the −13 position relative to the start codon indicates the P-site offset (34). As a further validation, all RPF samples showed a phase score >0.41, which is an indication of significant periodicity (35, 38). Clear groupings for all four biological replicates of fluconazole-treated and no drug Ribo-seq and RNA-seq samples were also observed in a principal component analysis (Fig. S1D). An analysis of all pairwise combinations of Ribo-seq (Fig. S2) and RNA-seq (Fig. S3) samples demonstrated a strong correlation in read counts, indicating a high degree of consistency. Overall, these findings validated the quality of our ribosome profiling data set that was used to examine global alterations in *C. albicans* translation in response to fluconazole treatment.

**Fig 1:**
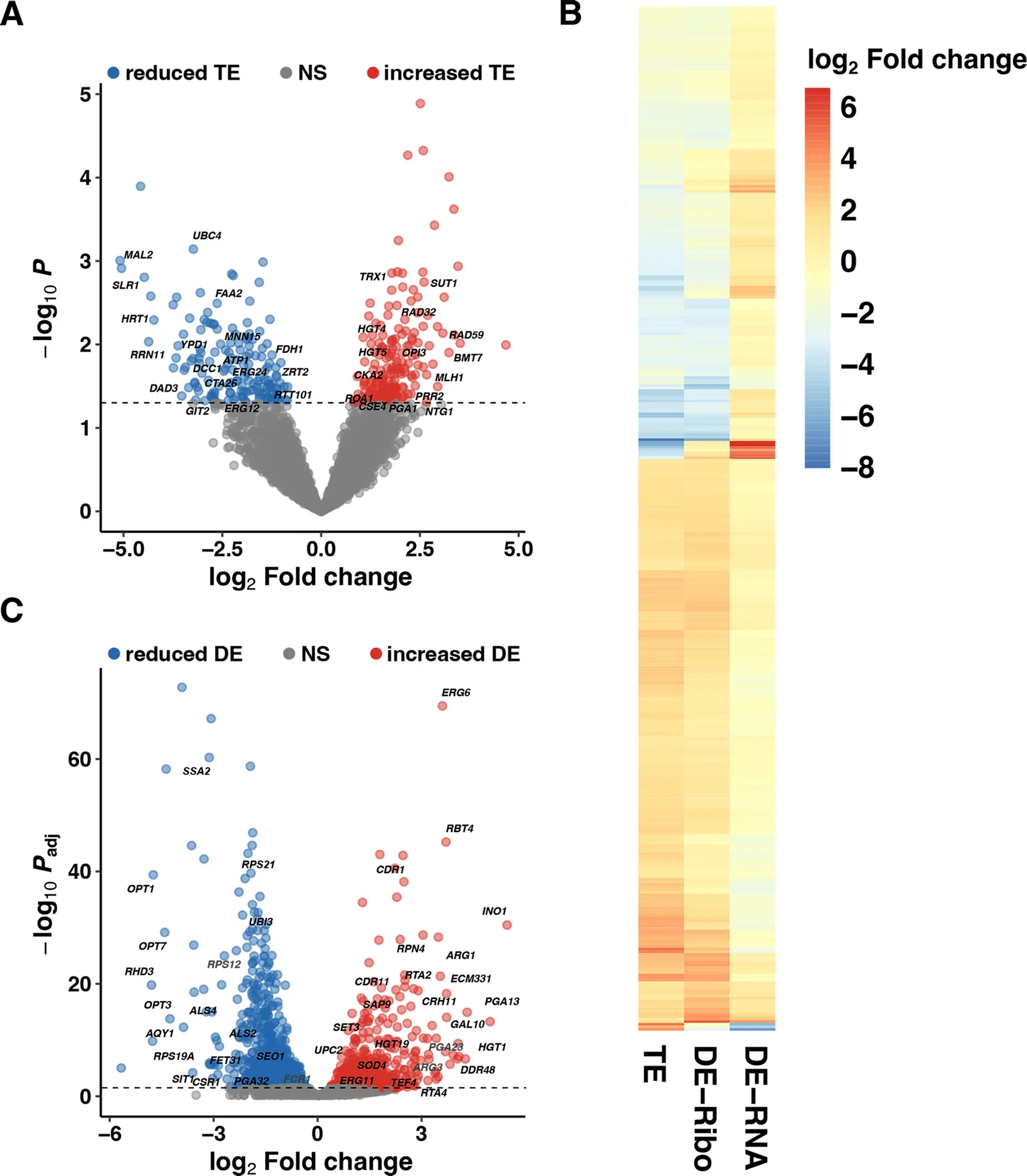
Identification of *C. albicans* genes showing alterations in translational efficiency and RNA differential expression (DE) in response to treatment with fluconazole. (**A**) Volcano plot showing genes with altered TE in the presence vs absence of fluconazole. Horizontal dashed line indicates *P* = 0.05 cutoff. (**B**) Heat map of genes with alterations in TE in the presence vs absence of fluconazole as defined in part (**A**) and Table 1. Corresponding differential expression values from both RNA-seq (DE-RNA) and Ribo-seq (DE-Ribo) in the presence vs absence of fluconazole are shown. (**C**) Volcano plot showing genes with altered RNA DE in the presence vs absence of fluconazole. Horizontal dashed line indicates *p*_adj_ = 0.05 cutoff. NS = nonsignificant.

### Ribosome profiling reveals significant differences between the translational and transcriptional responses of *C. albicans* to fluconazole treatment

**Fig 2:**
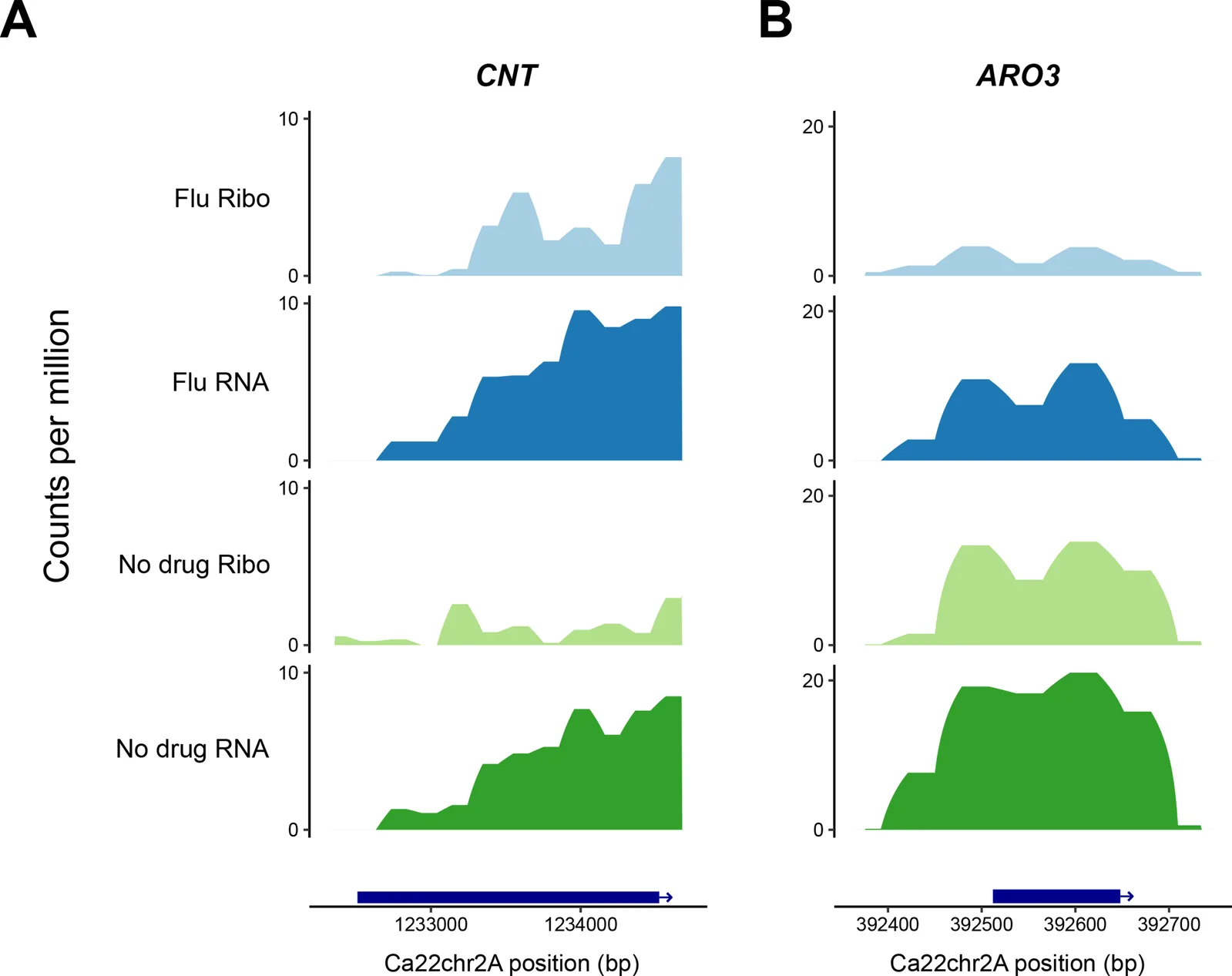
Example read coverage plots for *C. albicans* genes showing differential translational efficiency in response to fluconazole treatment. Normalized Ribo-seq and RNA-seq average read coverage across four replicates are shown for *CNT* (**A**), which shows increased TE, and *ARO3* (**B**), which shows reduced TE.

Using both Ribo-seq and RNA-seq reads, we determined alterations in translational efficiency (TE) in the presence vs absence of fluconazole for each gene in our data set. Overall, we identified 181 genes showing increased and 152 genes showing decreased TE (Fig. 1A; Table 1; Dataset S1). A high proportion of these genes exhibited large (≥4-fold) changes in TE (Table 1). Nearly, all genes with increased TE in response to fluconazole showed increased differential expression (DE) of Ribo-seq reads, while most genes with reduced TE showed reduced DE of Ribo-seq reads and several showed increased DE of RNA-seq reads (Fig. 1B). Example read count traces for one gene showing increased TE (*CNT*) and another gene showing reduced TE (*ARO3*) are presented in Fig. 2.

**TABLE 1:** Number of genes showing significantly altered translational efficiency and RNA differential expression during treatment of *C. albicans* with fluconazole

	≥2-fold	≥4-fold	≥8-fold
Number of genes showing increased TE^*a*^	181	59	9
Number of genes showing reduced TE^*a*^	152	81	34
Number of genes showing increased RNA DE^*b*^	337	104	28
Number of genes showing reduced RNA DE^*b*^	509	86	25

^*a*^ Fold changes are based on mean translational efficiency values in cells grown in YEPD + fluconazole vs YEPD + vehicle only control at 30°C at the 6-h time point, from four independent experiments (*n* = 4, TPM >1 in at least three replicates, *P* ≤ 0.05).

^*b*^ Fold changes are based on mean RNA differential expression values in cells grown in YEPD + fluconazole vs YEPD + vehicle only control at 30°C at the 6-h time point, from four independent experiments (*n* = 4, TPM >1 in at least three replicates, *P*_adj_ ≤ 0.05).

**Fig 3:**
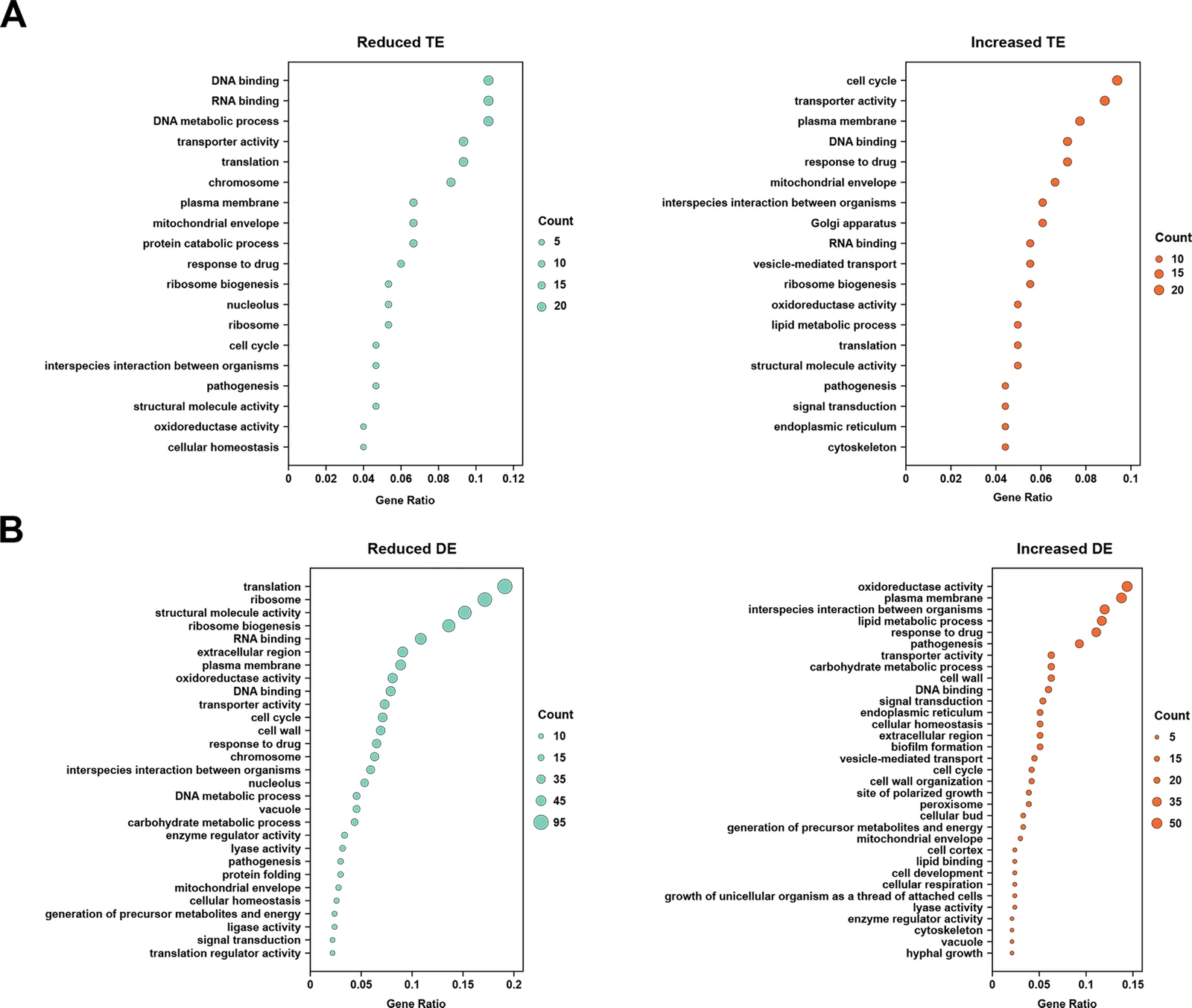
Gene ontology analysis of genes showing changes in TE and RNA DE in response to fluconazole. (**A**) Genes with significant alterations in TE, as defined in Table 1, were classified by GO terms using *C. albicans* GO Slim ontology (*Candida* Genome database, http://www.candidagenome.org/) and clusterProfiler (39). (**B**) Genes with significant alterations in RNA DE, as defined in Table 1, were classified by GO terms as described in part (**A**). Count = number of genes within each GO term.

Using the set of genes with increased TE in response to fluconazole treatment, a gene ontology (GO) analysis indicated a strong representation of gene categories associated with the cell cycle, plasma membrane, mitochondrial envelope, response to drug as well as transporters, and DNA-binding activity (Fig. 3A; Dataset S2). Interestingly, many genes involved in DNA repair showed elevated TE in response to fluconazole, including *RAD59*, *RAD32*, *NTG1,* and *MLH1* (Table 2; Dataset S1). Several membrane transporters also showed significantly increased TE, including *ROA1*, a PDR1-subfamily ABC transporter involved in sensitivity to azoles, the *HGT4* and *HGT5* glucose transporters, *MRS4*, a mitochondrial carrier family member involved in iron homeostasis, as well as *CNT*, an H^+^ nucleoside symporter. A number of genes involved in cell wall/cell membrane synthesis also demonstrated increased translation, including the *BMT7* β-mannosyltransferase, *OPI3* phosphatidylethanolamine *N*-methyltransferase, *PGA1* GPI-anchored protein, *ECM15,* and *PEX11* (Table 2; Dataset S1). Several genes associated with stress responses, including the *GPI14* glycosylphosphatidylinositol-α 1,4 mannosyltransferase I catalytic subunit and *TRX1* thioredoxin, involved in the response to reactive oxygen species, as well as *PLC1* phosphoinositide-specific phospholipase C, also showed increased TE. Finally, several signaling molecules, such as *CKA2*, a catalytic subunit of protein kinase CK2, which controls the calcineurin pathway to affect fluconazole sensitivity, *RAC1* G protein and *RAS1* GTPase, as well as the *SUT1* transcription factor, involved in sterol uptake, demonstrated elevated TE (Table 2; Dataset S1).

**TABLE 2:** Selected genes showing significantly increased translational efficiency during the *C. albicans* response to fluconazole treatment

Gene name^*a*^	Ref. no.	Description^*a*^	Fold change in TE^*b*^
*RAD59*	orf19.2630	Protein involved in homologous recombination and DNA break repair	7.7
*RAD32*	orf19.866	DNA polymerase with role in DNA repair; down-regulation associated with azole resistance	6.7
*PRR2*	orf19.1341	Putative serine/threonine protein kinase; mutation confers resistance to 5-flucytosine	6.4
*BMT7*	orf19.342	β-Mannosyltransferase; down-regulated in azole-resistant strain	6.0
*SUT1*	orf19.4342	Zn2-Cys6 transcription factor involved in sterol uptake	5.4
*NTG1*	orf19.5098	Protein with strong similarity to *Saccharomyces cerevisiae* DNA repair glycosylases	5.2
*MLH1*	orf19.4162	Putative mismatch repair protein	4.9
*GPI14*	orf19.2444	Catalytic subunit of glycosylphosphatidylinositol-α 1,4 mannosyltransferase, involved in GPI anchor biosynthesis; regulated under H_2_O_2_ stress conditions	4.4
*OPI3*	orf19.7446	Phosphatidylethanolamine *N*-methyltransferase; down-regulation correlated with fluconazole resistance; amphotericin B, caspofungin-repressed	3.8
*CSE4*	orf19.6163	Centromeric histone H3 variant; depleted from centromeres under chromosome destabilizing conditions, including treatment with fluconazole	3.7
*PGA1*	orf19.7625	Putative GPI-anchored protein; induced in cell wall regeneration; required for adhesion to host cells	3.7
*HGT4*	orf19.5962	Glucose and galactose sensor; roles in fermentation, filamentation, and virulence	3.7
*HGT5*	orf19.6005	Putative glucose transporter of major facilitator superfamily	3.6
*CKA2*	orf19.3530	Catalytic a subunit of protein kinase CK2; interaction with calcineurin pathway affects fluconazole sensitivity; important for virulence in oral candidiasis model	3.5
*CNT*	orf19.4118	CNT family H(+) nucleoside symporter	3.5
*TRX1*	orf19.7611	Thioredoxin; involved in response to reactive oxygen species; amphotericin B, caspofungin repressed	3.4
*ECM15*	orf19.7436.1	Predicted role in cell wall organization; caspofungin repressed	3.4
*PEX11*	orf19.1089	Putative peroxisomal membrane protein; role in fatty acid oxidation; induced by geldamycin	3.2
*ROA1*	orf19.4531	Putative PDR family ABC transporter involved in sensitivity to azoles	3.1
*ESP1*	orf19.3356	Putative caspase-like cysteine protease; mutation confers increased sensitivity to nocodazole	2.5

^*a*^ Gene names and descriptions based on *Candida* genome database annotation (http://www.candidagenome.org).

^*b*^ Indicates mean fold change in TE (*n* = 4, TPM >1 in at least 3 replicates, *P* ≤ 0.05) in cells grown in YEPD + fluconazole vs YEPD + vehicle only control at 30°C at the 6-h time point.

Among the set of genes with reduced TE in response to fluconazole, GO analysis indicated that DNA- and RNA-binding proteins showed the highest representation, followed by DNA metabolic processes, transporter activity and translation (Fig. 3A; Dataset S2). Several additional GO terms associated with protein synthesis, including ribosome biogenesis and ribosome, were also significantly represented in this gene set. Consistent with the down-regulation of protein synthesis, *RRN11*, a putative RNA polymerase I subunit; *NOP10*, a small nucleolar riboprotein; *RRP15*, a constituent of the pre-60S ribosomal particle; as well as *TIF34* and *TIF35*, putative translation initiation factors, all showed reduced TE (Dataset S1). Several genes associated with DNA replication, including *DAD3*, involved in chromosome segregation and *DCC1*, important for sister chromatid cohesion, demonstrated strongly reduced TE (Table 3; Dataset S1). Genes involved in ubiquitin-mediated protein degradation, such as *UBC4* and *RTT101*, also showed significantly reduced TE. Several signaling components, including *YPD1*, a phosphohistidine intermediate, and *NIK1*, a histidine kinase that controls *C. albicans* virulence and cell wall biosynthesis, demonstrated reduced TE in response to fluconazole. Interestingly, two genes in the ergosterol biosynthesis pathway, *ERG12* and *ERG24*, also showed a reduction in TE. *MAL2*, encoding a maltose-inducible α-galactosidase, and *SLR1*, a splicing factor involved in *C. albicans* filamentous growth and virulence, were among the genes most strongly reduced in TE upon fluconazole treatment (Table 3; Dataset S1).

**TABLE 3:** Selected genes showing significantly reduced translational efficiency during the *C. albicans* response to fluconazole treatment

Gene name^*a*^	Ref. no.	Description^*a*^	Fold change in TE^*b*^
*RBR1*	orf19.535	GPI-anchored cell wall protein required for filamentous growth at acidic pH	−34.1
*MAL2*	orf19.7668	α-Glucosidase; maltose induced; glucose repressed	−33.2
*SLR1*	orf19.1750	Protein similar to mammalian SR-like RNA splicing factor; involved in filamentous growth and virulence	−19.8
*HRT1*	orf19.233	Component of a nuclear ubiquitin-protein ligase complex involved in cell cycle control; induced by hydroxyurea	−13.4
*RRN11*	orf19.718	Putative RNA polymerase I subunit	−13.3
*DAD3*	orf19.3871	Subunit of the Dam1 (DASH) complex, which acts in chromosome segregation by coupling kinetochores to spindle microtubules	−10.2
*UBC4*	orf19.7571	Ortholog(s) have protein binding, bridging, ubiquitin binding, ubiquitin-conjugating enzyme activity	−9.4
*DCC1*	orf19.7083	Protein with a predicted role in sister chromatid cohesion and telomere length maintenance	−9.1
*GIT2*	orf19.1978	Putative glycerophosphoinositol permease; fungal-specific	−8.6
*YPD1*	orf19.4443	Phosphohistidine intermediate protein in a phosphorelay signal transduction pathway	−8.5
*FAA2*	orf19.7379	Putative acyl CoA synthetase	−7.8
*MNN15*	orf19.753	Putative α-1,3-mannosyltransferase; predicted role in protein O-linked glycosylation	−6.5
*ATP1*	orf19.6854	ATP synthase alpha subunit; ciclopirox, ketoconazole, flucytosine induced; Efg1, caspofungin repressed	−5.4
*CTA26*	orf19.7680	Putative transcription factor/activator	−4.9
*NOP10*	orf19.596	Small nucleolar ribonucleoprotein; flucytosine induced	−3.7
*ERG12*	orf19.4809	Ortholog(s) have mevalonate kinase activity and role in ergosterol biosynthetic process	−3.7
*FDH1*	orf19.638	Formate dehydrogenase; oxidizes formate to CO_2_; induced by macrophages; fluconazole-repressed	−3.5
*ERG24*	orf19.1598	C-14 sterol reductase, has a role in ergosterol biosynthesis	−3.3
*ZRT2*	orf19.1585	Zinc transporter; ciclopirox olamine, fluconazole, alkaline repressed; transcript induced by amphotericin B; interaction with macrophages	−3.0
*NIK1*	orf19.5181	Histidine kinase involved in a two-component signaling pathway that regulates cell wall biosynthesis; required for wild-type virulence in mouse systemic infection	−2.9
*RTT101*	orf19.2440	Putative cullin subunit of E3 ubiquitin ligase complex; involved in response to DNA damage	−2.8

^*a*^ Gene names and descriptions based on *Candida* genome database annotation (http://www.candidagenome.org).

^*b*^ Indicates mean fold change in TE (*n* = 4, TPM >1 in at least three replicates, *P* ≤ 0.05) in cells grown in YEPD + fluconazole vs YEPD + vehicle only control at 30°C at the 6-h time point.

Because standard RNA-seq analysis is required for ribosome profiling, we were also able to re-examine the transcriptional response to fluconazole using RNA-seq, rather than DNA microarrays, which had been used in most previous studies on the response of *C. albicans* to antifungal treatment. We identified 337 genes with ≥2-fold increased RNA DE and 509 genes with ≥2-fold reduced RNA DE (Fig. 1C; Table 1; Dataset S1). GO analysis indicated that gene categories associated with oxidoreductase activity, plasma membrane, interspecies interaction between organisms, lipid metabolic processes, response to drug, pathogenesis, and transporter activity showed the highest representation in the set of genes with increased transcript expression (Fig. 3B; Dataset S2). As expected, and as previously observed (32, 33), a large number of genes involved in ergosterol biosynthesis were induced (*ERG1*, *ERG3*, *ERG4*, *ERG6*, *ERG7*, *ERG11*, *ERG25*, *ERG27*, *ERG251*) (Dataset S1). Two putative ABC transporters, *CDR11* and *SNQ2*, associated with drug efflux, were also in this group, in addition to cell wall genes (*PGA23*, *ECM331*, *WSC1*), stress response genes (*YHB1*, *SOD4*, *CRZ1*), and several transcription factors (*UPC2*, *TAC1*, *BCR1*, *STP4*, *RIM101, RFX1, RGT1, ZCF32*) (Dataset S1). *UPC2* has previously been shown to be important for induction of ergosterol biosynthesis genes and sterol uptake (40), *TAC1* is involved in the induction of drug transporters, *BCR1* is important for azole resistance, and *STP4* is associated with the response to the echinocandin drug caspofungin. Interestingly, however, the other transcription factors (*RIM101*, *RFX1, RGT1*) are involved in diverse *C. albicans* processes such as alkaline-induced filamentous growth, DNA damage responses, virulence, glucose transport, and biofilm formation; to our knowledge, *RFX1* has not previously been associated with the response to antifungal treatment.

Based on our GO analysis, the set of genes down-regulated at the RNA level in response to fluconazole showed enrichment for several gene categories associated with protein synthesis, including translation, ribosome, and ribosome biogenesis (Fig. 3B; Dataset S2). Other gene categories strongly represented included plasma membrane as well as structural molecule and RNA-binding activities. We also noted that a significant number of genes involved in amino acid transport showed reduced transcript levels in response to fluconazole, including four oligopeptide transporters (*OPT1*, *OPT2*, *OPT3*, *OPT7*), the *GAP2* amino acid permease, and the *AAT22* aspartate aminotransferase (Dataset S1). Interestingly, two members of the *ALS* family of adhesins (*ALS2* and *ALS4*), as well as *SSA2*, a *HSP70* family chaperone, also showed reduced transcript levels (Dataset S1). Similar, but not identical, sets of genes showed altered RNA DE in response to fluconazole in a more recent RNA-seq-based study (33).

Overall, there were both similarities and differences in the most highly represented gene categories under translational vs transcriptional control in response to fluconazole. For example, a larger fraction of cell cycle genes appears to be translationally vs transcriptionally upregulated, whereas both plasma membrane and transporter gene categories were highly represented among the sets of genes showing increased translation and transcription (Fig. 3). Interestingly, however, we observed large differences in the specific genes showing altered translation vs transcription in response to fluconazole. Of the 181 genes showing significantly increased TE, only 9 (5%) were also up-regulated at the transcriptional level and 30 (17%) were down-regulated (Dataset S1). Down-regulated genes included several involved in DNA damage repair (*RAD32*, *MLH1*), signaling (*RAC1*, *PRR2*), and translation (*RPL39*, *TEF1*, *MRP17*, *TIF5*, *ANB1*). Of the 152 genes showing significantly reduced TE, only 6 (4%) showed reduced transcription, whereas 16 (11%) showed increased transcription (including *ERG24*, involved in ergosterol biosynthesis) (Dataset S1).

### Identification of novel *C. albicans* transcripts showing ribosome occupancy

**Fig 4:**
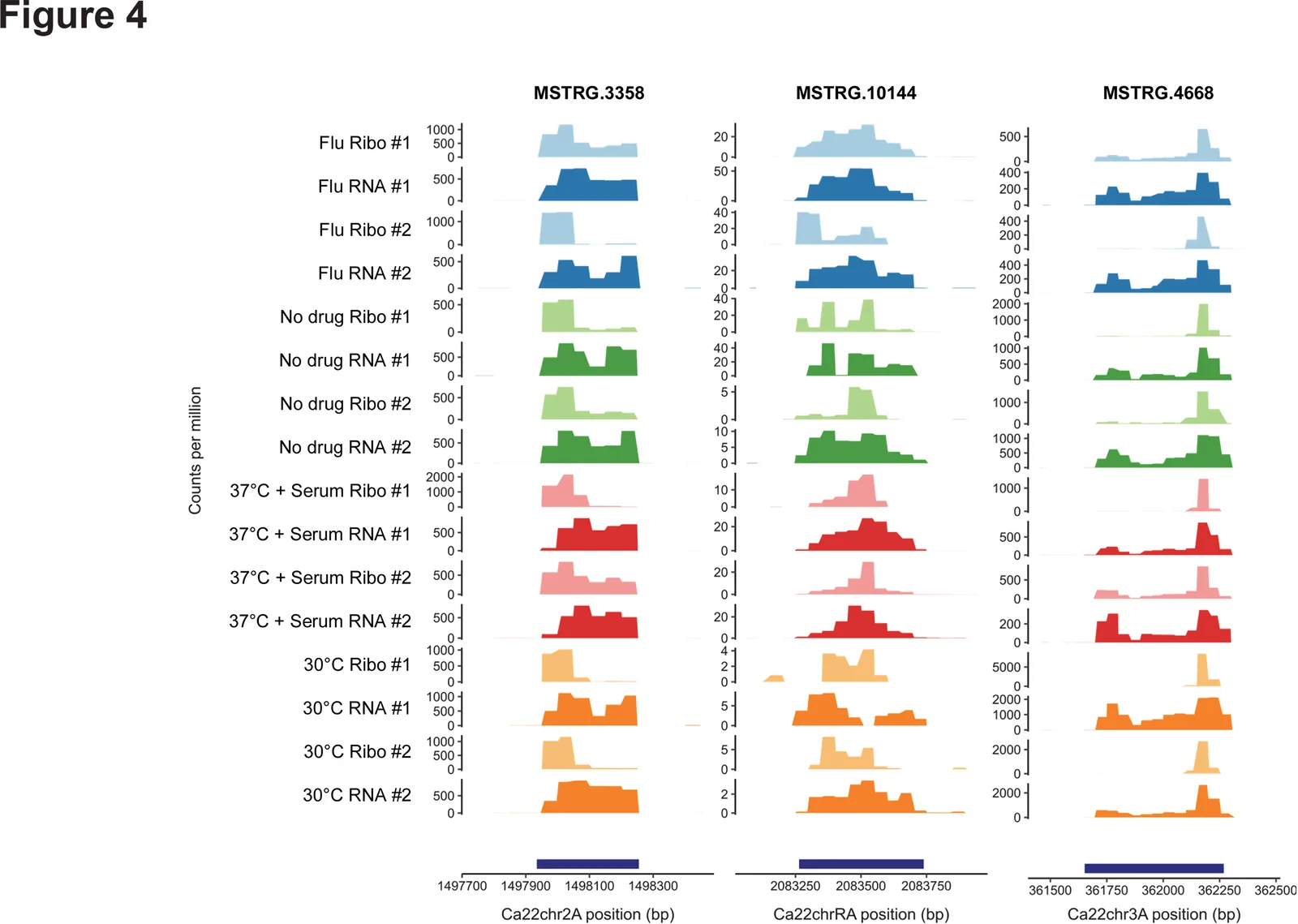
Ribosome occupancy in novel *C. albicans* transcripts. Normalized Ribo-seq and RNA-seq counts for three novel transcripts are shown in the presence and absence of fluconazole (Flu) as well as under filament-inducing (37°C + serum) vs non-inducing (30°C) conditions (data for morphology experiment are from reference (35)). Two independent biological replicates are shown for each condition.

We have previously used ribosome profiling to identify several actively translating novel *C. albicans* genes with a recently developed bioinformatic pipeline that incorporates both RNA-seq reads and three-nucleotide periodicity of Ribo-seq reads (35). In our current study, we identified three novel *C. albicans* transcripts that did not map to known coding regions, which we have named *MSTRG.3358*, *MSTRG.10144,* and *MSTRG.4668*. The transcripts, which range from 321 to 670 bp in length and are not located immediately adjacent to known coding sequences (Dataset S3), showed strong 3-nucleotide periodicity (phase score >0.41) of RPFs but, at least initially, did not appear to contain genes based on the presence of potential stop codons. However, we cannot exclude the possibility of short open reading frames. Interestingly, all three transcripts showed a significant number of both RNA-seq and Ribo-seq read counts (Fig. 4; Dataset S3). Increased read count densities were consistently observed in these regions across all four biological replicates. In examining data for our previous ribosome profiling study on *C. albicans* morphology (35), we also consistently observed significant RNA-seq and Ribo-seq reads for these regions in all biological replicates as well. However, the read counts do not appear to vary in fluconazole vs no drug and 37°C + serum (filaments) vs 30°C (yeast) samples (Fig. 4; Dataset S3).

## DISCUSSION

While several previous studies have examined the genome-wide transcriptional response of fungal pathogens to antifungal treatment, this study is the first to examine the global translational response. We observed several similarities in the categories of genes controlled by *C. albicans* at both the transcriptional and translational levels. More specifically, gene categories associated with the cell wall/cell membrane and transport were highly represented in the sets of genes showing elevated translational efficiency and transcript levels (Fig. 3). In addition, gene categories associated with protein synthesis were highly represented among the sets of genes strongly down-regulated at both the translational and transcriptional levels in response to fluconazole treatment; this finding is consistent with previous studies indicating a widespread reduction in protein synthesis following antifungal treatment (32, 33) and is most likely part of a general stress response. However, there were also significant differences in translational vs transcriptional regulation of specific gene categories. For example, genes associated with the cell cycle and DNA damage repair were more highly represented in the increased TE vs RNA DE group (Fig. 3). In addition, genes associated with DNA-binding proteins and DNA metabolic processes were more highly represented in the reduced TE vs RNA DE group. Differences in translational vs transcriptional regulation in response to fluconazole treatment became more apparent at the level of specific genes. Strikingly, only a very small fraction of genes showing an increase or decrease in TE also showed a similar change in transcription. In fact, a significantly larger number of genes with increased TE showed reduced RNA DE and vice versa. These findings are consistent with our previous observations (35) and suggest that the *C. albicans* response to fluconazole is under widespread translational control that does not simply parallel the transcriptional response.

Our analysis has shown that a variety of genes involved in processes previously associated with the response to fluconazole are specifically up-regulated at the translational, but not transcriptional, level. These genes include the *ROA1* PDR-subfamily ABC transporter as well as several genes involved in cell wall/cell membrane synthesis (*BMT7*, *OPI1*, *PGA1*, *ECM15*, *PEX11*) and stress responses (*GPI14*, *TRX1, PLC1*). Interestingly, down-regulation of transcript levels for several of these genes, including *BMT7* and *OPI3,* was correlated with fluconazole resistance (41, 42). *TRX1* and *ECM15* transcripts were also repressed in response to treatment with other antifungals (30). In these cases, translational up-regulation may serve to fine tune protein levels for an optimal response to drug treatment. These findings are also consistent with our observations that transcriptional gene expression patterns do not necessarily parallel translational patterns with respect to antifungal treatment. Translational up-regulation of genes associated with DNA repair may be part of a larger response to cellular stresses, which can include DNA-damaging agents. Interestingly, in addition to the *ROA1* drug transporter, we also observed increased translation of two glucose transporters, as well as a mitochondrial membrane transporter important for iron homeostasis. These findings suggest that *C. albicans* uses specific translational mechanisms to promote the acquisition of key nutrients under drug stress conditions. Altered translation of multiple signaling molecules (*RAC1*, *RAS, YPD1, NIK1*) also suggests that specific translational mechanisms control signal transduction pathways that may be part of the response to fluconazole.

Overall, our RNA-seq analysis identified significantly fewer *C. albicans* genes showing altered transcription in response to fluconazole compared to a previous DNA microarray-based study (32). In addition to the method of analysis (RNA-seq vs DNA microarray), these discrepancies could be attributed to differences in fluconazole concentration, exposure time, growth medium, as well as statistical cut-offs that were used. Both the Lepak et al. study (32) and our current study show that genes associated with cell membrane/cell wall and stress responses are up-regulated and genes associated with protein synthesis are down-regulated at the transcriptional level. However, as expected for RNA-seq, our study identified additional genes associated with these processes that were not previously identified in the Lepak et al. study (32). We also show that several transcription factors are transcriptionally induced in response to fluconazole, and two members of the *ALS* adhesin gene family are transcriptionally down-regulated. These findings, which are mostly consistent with those of a more recent RNA-seq study (33), suggest that additional regulatory pathways and processes are associated with the transcriptional response to drug treatment. Multiple genes associated with amino acid transport, including oligopeptide transporters, also showed significant transcriptional down-regulation in response to fluconazole, which is consistent with our current observation and previous observations of a strong reduction in protein synthesis (32, 33). Finally, similar to previous studies (30 – 33), we observed transcriptional induction of many genes important for ergosterol biosynthesis. Interestingly, two genes in this pathway (*ERG12*, *ERG24*) showed reduced TE, one of which was also induced at the transcriptional level. These findings suggest the presence of novel translational mechanisms that may be specifically responsible for regulating ergosterol biosynthesis in response to fluconazole treatment. Overall, our study suggests that the response of *C. albicans*, and most likely additional human fungal pathogens, to antifungal treatment is under widespread translational control that does not necessarily parallel known transcriptional mechanisms. Ultimately, components of several of these translational mechanisms may serve as targets for the development of novel antifungal therapies.

Our identification of novel transcribed regions of the *C. albicans* genome with strong ribosome occupancy and 3-nucleotide periodicity suggests that they encode proteins. Initially, this conclusion seemed paradoxical given that these transcripts contain a significant number of stop codons. However, a previous ribosome profiling study made a very similar observation with respect to certain *linc* RNAs in mice (43). Using harringtonine as a protein synthesis inhibitor to accurately map specific translation start sites, the authors demonstrated that these RNAs were polycistronic, with multiple small coding sequences. While our current study did not use harringtonine, polycistronic mRNAs appear to represent a viable explanation. Additional ribosome profiling studies, which are able to determine translation start sites with greater accuracy, could be performed in the future to test this intriguing hypothesis.

## MATERIALS AND METHODS

### *Candida albicans* strain and growth conditions

For ribosome profiling experiments, a saturated overnight culture of *C. albicans* wild-type strain SC5314 was grown in YEPD (yeast extract‐peptone‐dextrose) medium at 30°C and 200 rpm. The culture was diluted in 50-mL YEPD medium and incubated until reaching an OD_600_ of 1.0. The culture was further diluted to an OD_600_ of 0.025 in 400-mL fresh YEPD and grown to OD_600_ = 0.050. At this point, 200 mL of culture was transferred to two 1-L flasks. Fluconazole was added to one flask at 1 µg/mL, and an equivalent volume of DMSO was added to the second flask as a vehicle-only control. Both flasks were incubated at 30°C for 6 h, which we had previously determined as the fluconazole IC_50_. To confirm that a 50% reduction in cell count occurred due to fluconazole treatment, OD_600_ of both cultures was measured and compared.

### Cell extract preparation and ribosome profiling

Cells were recovered by filtration at room temperature, rapidly scraped off the filter paper with a cell scraper, placed into ice-cold lysis buffer (1× Yeast polysome buffer (Illumina), 10% Triton X-100 (Sigma), 10 mg/mL GMPPNP (Sigma), 10 mg/mL Blasticidin S (Invivogen)) and homogeneously mixed, followed by snap freezing in liquid nitrogen. Cell lysates were prepared, and ribosome profiling was performed using four biological replicates as we have described previously (35).

### Ribo-seq and RNA-seq data analysis

Assessment of read quality, adapter trimming, GO analysis, as well as identification of novel ORFs and genes showing altered RNA DE and TE were performed as previously described (35). TrimGalore (v 0.4.3) (44) was used to trim adaptor and low-quality (phred quality score <5) score bases and retained reads were at least 20 nucleotides long for both RNA-seq and Ribo-seq libraries. To map the trimmed reads, we used STAR (v2.5.2b) (45). We allowed each alignment to have a maximum of two mismatched bases. Reads were first aligned to non-mRNA reference (C_albicans_SC5314_version_A22-s07-m01-r27_other_features_no_introns.fasta) obtained from *Candida* genome database, (http://www.candidagenome.org/). The unmapped reads to the non-mRNA reference were then mapped to Assembly 22 reference using GTF s07-m01-r27 as the guiding annotation. For both Ribo-seq and RNA-seq libraries, we performed gene-level counting using featureCounts (v1.6.4) (46). To account for the diploid *C. albicans* Assembly 22, we first counted reads for each allele separately and then took their sum as the input for differential expression and translational efficiency analysis. Ribotricer (v1.3.2) (38) was used to perform periodicity analysis of the Ribo-seq data. We defined an “ORF” as any sequence with a start codon differing from AUG by at most one nucleotide as well as possessing an in-frame stop codon and then used ribotricer to generate a periodicty score for each of these ORFs. An ORF was annotated to be “translating” if it displayed a phase score >0.41. DESeq2 (v1.38.0) (47) was used to perform DE analysis of the RNA-seq data. We defined genes to be differentially expressed if their absolute log_2_ fold-change was at least 1 with an FDR-adjusted *P*-value of at least 0.05 as long as the associated transcripts per million (TPM) was >1 in at least three out of four biological replicates. To perform differential TE analysis, we used riborex (v2.4.0) (48) using only genes that had at least one read count per replicate across both fluconazole-treated and no drug Ribo-seq and RNA-seq samples. Genes were defined to be showing differential TE if their TPM was greater than 1 in at least three out of four biological replicates and their absolute fold-change on a log_2_ scale was at least 1 with a corresponding non-adjusted *P*-value of at least 0.05. To perform GO analyses, we used clusterProfiler (v4.3.0) (39), using the GO slim ontology file available from *Candida* Genome database, (http://www.candidagenome.org/). Genes used for GO analysis showed a TPM > 1 in at least three out of four biological replicates.

## Data Availability

All raw and processed Ribo-seq and RNA-seq data from this study are available at the NCBI Gene Expression Omnibus (GEO) database under accession number GSE227590. Custom scripts used to generate the manuscript data are available at Github.

## SUPPLEMENTAL MATERIAL

Dataset S1 spectrum.02572-23-s0001.xlsx RNA differential gene expression (DE) and translational efficiency (TE) data for *C. albicans* cells grown in the presence vs absence of fluconazole.

Dataset S2 spectrum.02572-23-s0002.xlsx Gene Ontology (GO) data for *C. albicans* genes showing altered RNA differential gene expression (DE) and translational efficiency (TE) in response to treatment with fluconazole.

Dataset S3 spectrum.02572-23-s0003.xlsx TPM counts for novel *C. albicans* transcribed regions showing ribosome occupancy.

Supplemental material spectrum.02572-23-s0004.pdf Supplemental Figures S1 to S3.

ASM does not own the copyrights to Supplemental Material that may be linked to, or accessed through, an article. The authors have granted ASM a non-exclusive, world-wide license to publish the Supplemental Material files. Please contact the corresponding author directly for reuse.
