# Correction for Choudhary et al., “Genome-wide translational response of *Candida albicans* to fluconazole treatment”

**DOI:** 10.1128/spectrum.00678-24

**Published:** 2024-04-03

**Authors:** Saket Choudhary, Vasanthakrishna Mundodi, Andrew D. Smith, David Kadosh

## AUTHOR CORRECTION

Volume 11, no. 5, e02572-23, 2023, https://doi.org/10.1128/spectrum.02572-23. Page 12: “lysis buffer (1× Yeast polysome buffer (Illumina), 10% Triton X-100 (Sigma), 10 mg/mL GMPPNP (Sigma), 10 mg/mL Blasticidin S (Invivogen))” should read “lysis buffer [1× yeast polysome buffer (Illumina), 1% Triton X-100 (Sigma), 50 μg/mL GMPPNP (Sigma), 10 μg/mL Blasticidin S (InvivoGen)].”

