# Supplementary material for: Genome-wide translational response of Candida albicans to fluconazole treatment
Source: Microbiol Spectr. 2023 Aug 23;11(5):e02572-23. doi: 10.1128/spectrum.02572-23 (PMC10580883; doi:10.1128/spectrum.02572-23)
Supplement: Supplemental material — Supplemental Figures S1 to S3. [file spectrum.02572-23-s0004.pdf]

SUPPLEMENTAL MATERIAL

Supplemental Figure Legends

Figure S1. Data quality analysis for ribosome profiling experiments. (A) Example read length distribution plot of ribosome-protected fragments (RPFs) generated from a sample grown in the absence of fluconazole. (B) General correlation between RNA-seq and Ribo-seq read counts as demonstrated by a scatter plot using data from the sample described in part (A). (C) Reading frame enrichment metagene plots generated using a 30 nt. read length from the sample described in part (A). The plots show normalized read counts for each position relative to the start codon (indicated by position 0) for genes. (D) Principal Components Analysis (PCA) for Ribo-seq and RNA-seq samples grown in the presence and absence of fluconazole (showing all four biological replicates). TPM = transcripts per million, nt = nucleotide.

Figure S2. Consistency among Ribo-seq biological replicates. Scatter plots showing a strong correlation in Ribo-seq TPM counts for all four biological replicates in the presence and absence of fluconazole.

Figure S3. Consistency among RNA-seq biological replicates. Scatter plots showing a strong correlation in RNA-seq TPM counts for all four biological replicates in the presence and absence of fluconazole.

Supplemental Datasets

Dataset S1. RNA differential gene expression (DE) and translational efficiency (TE) data for *C. albicans* cells grown in the presence vs. absence of fluconazole. Gene names and descriptions are based on annotations in the *Candida* Genome Database (http://www.candidagenome.org).

Dataset S2. Gene Ontology (GO) data for *C. albicans* genes showing altered RNA differential gene expression (DE) and translational efficiency (TE) in response to treatment with fluconazole.

Dataset S3. TPM counts for novel *C. albicans* transcribed regions showing ribosome occupancy.

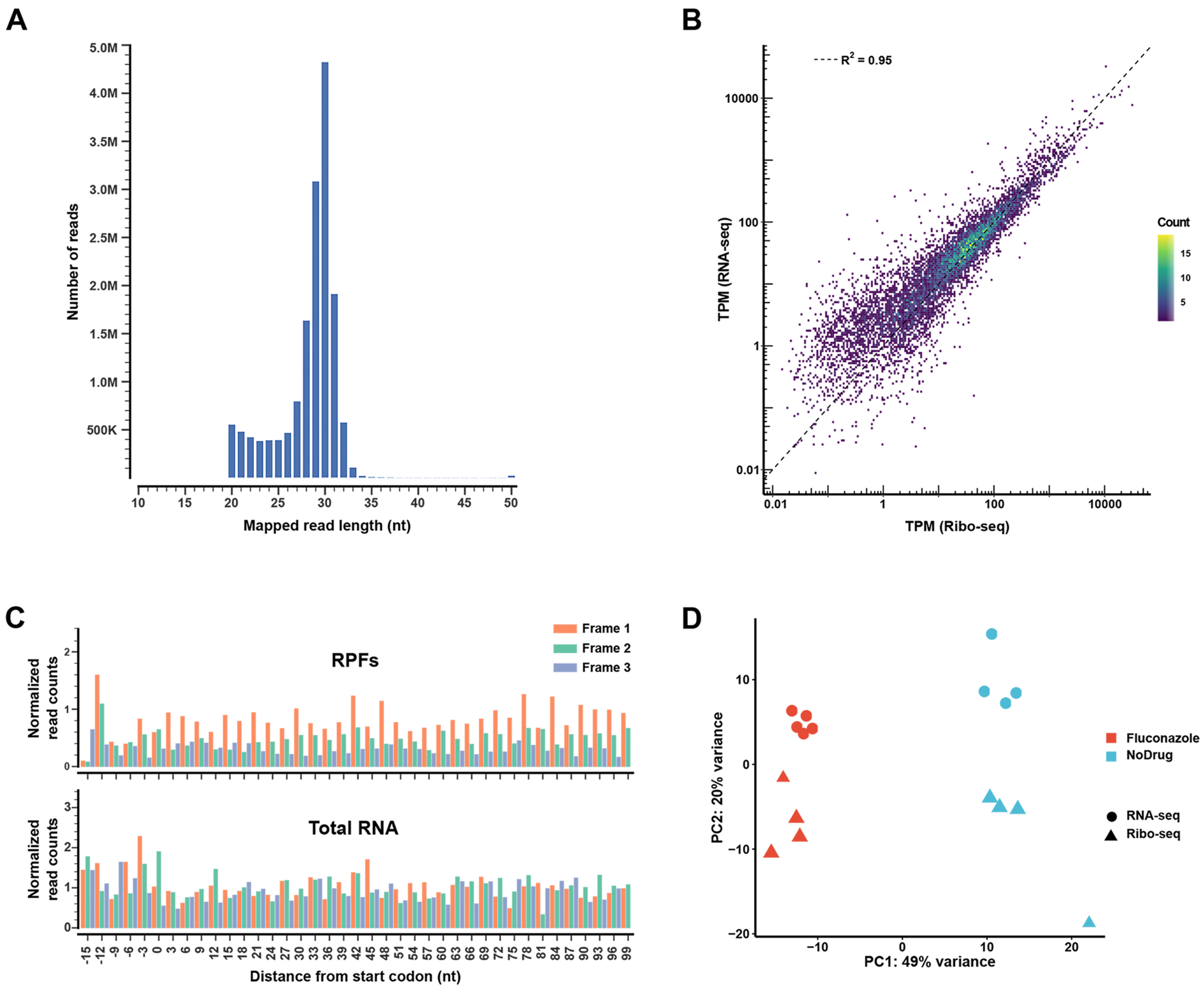
A
5.0M
4.0M
3.5M
3.0M
2.5M
2.0M
1.5M
1.0M
500K
Number of reads
10
15
20
25
30
35
40
45
50
Mapped read length (nt)
B
R^2 = 0.95
10000
100
1
0.01
TPM (RNA-seq)
0.01
1
100
10000
TPM (Ribo-seq)
Count
15
10
5
C
Frame 1
Frame 2
Frame 3
RPFs
Normalized read counts
Total RNA
Distance from start codon (nt)
D
10
0
−10
−20
PC2: 20% variance
−10
0
10
20
PC1: 49% variance
Fluconazole
NoDrug
RNA-seq
Ribo-seq

Figure S1

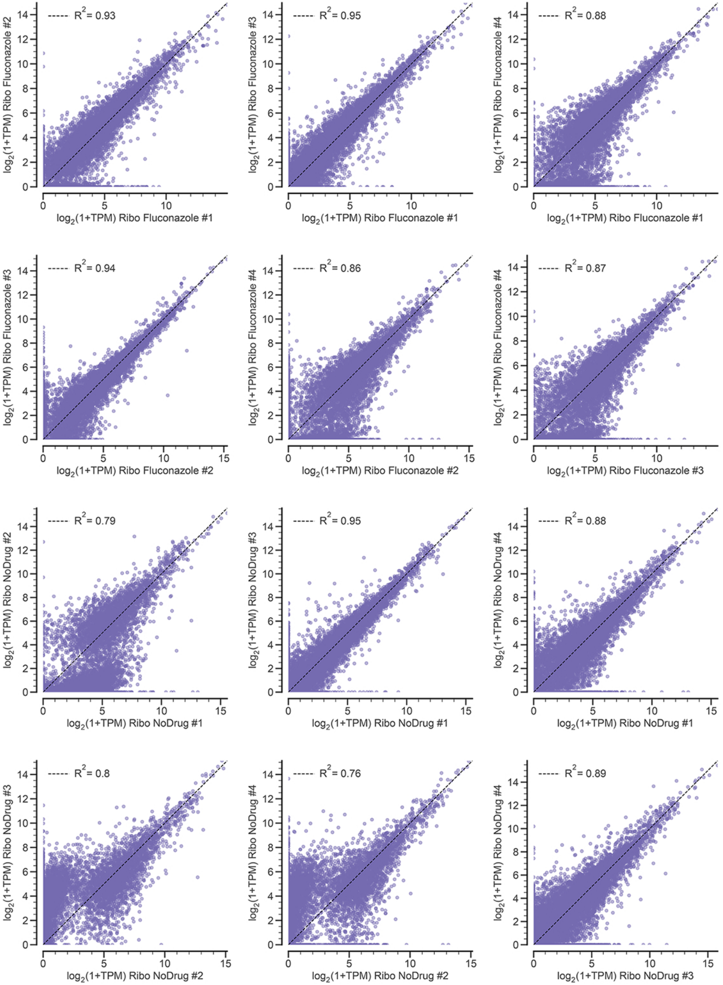

R2 = 0.93
log2(1+TPM) Ribo Fluconazole #2
log2(1+TPM) Ribo Fluconazole #1
R2 = 0.95
log2(1+TPM) Ribo Fluconazole #3
log2(1+TPM) Ribo Fluconazole #1
R2 = 0.88
log2(1+TPM) Ribo Fluconazole #4
log2(1+TPM) Ribo Fluconazole #1
R2 = 0.94
log2(1+TPM) Ribo Fluconazole #3
log2(1+TPM) Ribo Fluconazole #2
R2 = 0.86
log2(1+TPM) Ribo Fluconazole #4
log2(1+TPM) Ribo Fluconazole #2
R2 = 0.87
log2(1+TPM) Ribo Fluconazole #4
log2(1+TPM) Ribo Fluconazole #3
R2 = 0.79
log2(1+TPM) Ribo NoDrug #2
log2(1+TPM) Ribo NoDrug #1
R2 = 0.95
log2(1+TPM) Ribo NoDrug #3
log2(1+TPM) Ribo NoDrug #1
R2 = 0.88
log2(1+TPM) Ribo NoDrug #4
log2(1+TPM) Ribo NoDrug #1
R2 = 0.8
log2(1+TPM) Ribo NoDrug #3
log2(1+TPM) Ribo NoDrug #2
R2 = 0.76
log2(1+TPM) Ribo NoDrug #4
log2(1+TPM) Ribo NoDrug #2
R2 = 0.89
log2(1+TPM) Ribo NoDrug #4
log2(1+TPM) Ribo NoDrug #3

Figure S2

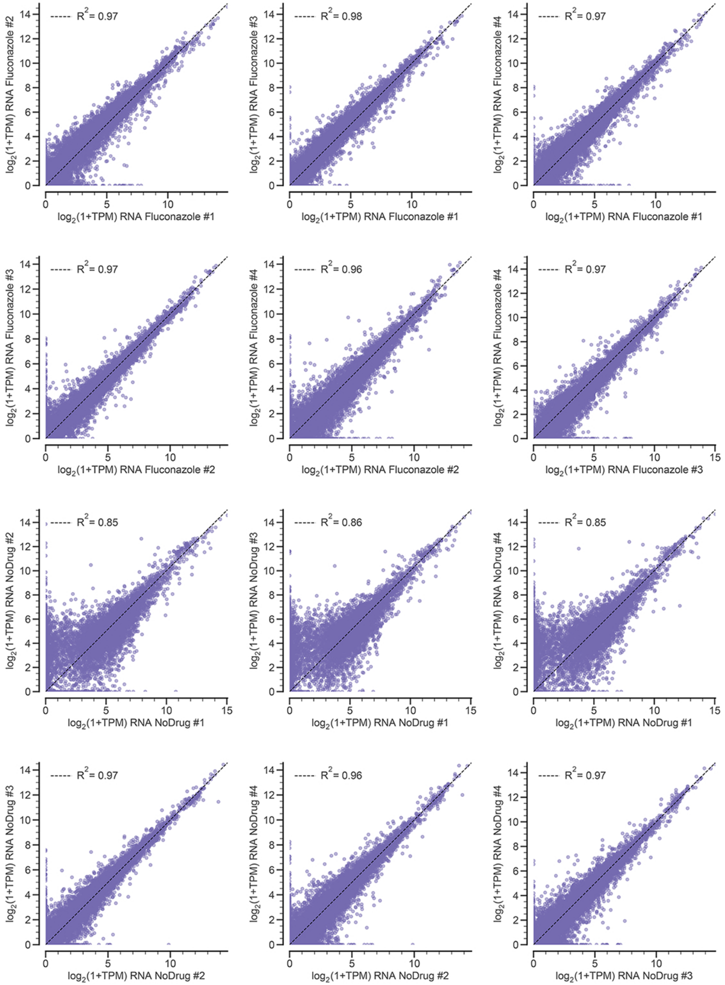
R^2 = 0.97
log2(1+TPM) RNA Fluconazole #2
log2(1+TPM) RNA Fluconazole #1
R^2 = 0.98
log2(1+TPM) RNA Fluconazole #3
log2(1+TPM) RNA Fluconazole #1
R^2 = 0.97
log2(1+TPM) RNA Fluconazole #4
log2(1+TPM) RNA Fluconazole #1
R^2 = 0.97
log2(1+TPM) RNA Fluconazole #3
log2(1+TPM) RNA Fluconazole #2
R^2 = 0.96
log2(1+TPM) RNA Fluconazole #4
log2(1+TPM) RNA Fluconazole #2
R^2 = 0.97
log2(1+TPM) RNA Fluconazole #4
log2(1+TPM) RNA Fluconazole #3
R^2 = 0.85
log2(1+TPM) RNA NoDrug #2
log2(1+TPM) RNA NoDrug #1
R^2 = 0.86
log2(1+TPM) RNA NoDrug #3
log2(1+TPM) RNA NoDrug #1
R^2 = 0.85
log2(1+TPM) RNA NoDrug #4
log2(1+TPM) RNA NoDrug #1
R^2 = 0.97
log2(1+TPM) RNA NoDrug #3
log2(1+TPM) RNA NoDrug #2
R^2 = 0.96
log2(1+TPM) RNA NoDrug #4
log2(1+TPM) RNA NoDrug #2
R^2 = 0.97
log2(1+TPM) RNA NoDrug #4
log2(1+TPM) RNA NoDrug #3

Figure S3
